# An exploratory *in silico* comparison of open-source codon harmonization tools

**DOI:** 10.1186/s12934-023-02230-y

**Published:** 2023-11-06

**Authors:** Thomas Willems, Wim Hectors, Jeltien Rombaut, Anne-Sofie De Rop, Stijn Goegebeur, Tom Delmulle, Maarten L. De Mol, Sofie L. De Maeseneire, Wim K. Soetaert

**Affiliations:** Centre for Industrial Biotechnology and Biocatalysis (InBio.be), Department of Biotechnology, Faculty of Bioscience Engineering, Ghent University, Coupure Links 653, Ghent, 9000 Belgium

**Keywords:** Synthetic Biology, Codon usage Bias, Codon Harmonization, EuGene, Galaxy, CodonWizard, CHARMING

## Abstract

**Background:**

Not changing the native constitution of genes prior to their expression by a heterologous host can affect the amount of proteins synthesized as well as their folding, hampering their activity and even cell viability. Over the past decades, several strategies have been developed to optimize the translation of heterologous genes by accommodating the difference in codon usage between species. While there have been a handful of studies assessing various codon optimization strategies, to the best of our knowledge, no research has been performed towards the evaluation and comparison of codon harmonization algorithms. To highlight their importance and encourage meaningful discussion, we compared different open-source codon harmonization tools pertaining to their *in silico* performance, and we investigated the influence of different gene-specific factors.

**Results:**

In total, 27 genes were harmonized with four tools toward two different heterologous hosts. The difference in %MinMax values between the harmonized and the original sequences was calculated (ΔMinMax), and statistical analysis of the obtained results was carried out. It became clear that not all tools perform similarly, and the choice of tool should depend on the intended application. Almost all biological factors under investigation (GC content, RNA secondary structures and choice of heterologous host) had a significant influence on the harmonization results and thus must be taken into account. These findings were substantiated using a validation dataset consisting of 8 strategically chosen genes.

**Conclusions:**

Due to the size of the dataset, no complex models could be developed. However, this initial study showcases significant differences between the results of various codon harmonization tools. Although more elaborate investigation is needed, it is clear that biological factors such as GC content, RNA secondary structures and heterologous hosts must be taken into account when selecting the codon harmonization tool.

**Supplementary Information:**

The online version contains supplementary material available at 10.1186/s12934-023-02230-y.

## Background

The ability to introduce and express heterologous gene sequences in microorganisms, enabled by synthetic biology, is regarded as a cornerstone of modern biotechnology. It has contributed to the successful expansion of biotechnological processes by allowing the development of well-characterized microbial cell factories that can adopt recombinant DNA to acquire novel functionalities. This has led to breakthroughs in various areas, such as the production of platform chemicals through carbon capture utilization [1, 2] or the more cost-efficient production of therapeutics and vaccines [3, 4], as well as of industrial enzymes, all of which are being increasingly applied in food processing, detergent, and biofuel industries [5–7]. To obtain these functionalities, microbial cell factories often require rewiring, altering or finetuning of their metabolism, warranting the introduction of recombinant genes.

Here, the efficiency of gene expression as well as the metabolic burden associated with it are two major factors affecting the product yield of microbial cell factories [8]. Although often overlooked, a key element herein is the difference in codon usage between the natural host and the intended microbial cell factory. As 61 codons encode only 20 amino acids, the genetic code is considered redundant. However, synonymous codons are unequally distributed in genomes. While historically regarded as functionally neutral, evidence now reveals that synonymous codon usage is nonrandom and affects multiple facets of functional protein biosynthesis, which spurred the development of codon optimization approaches [9]. Rare codons were initially presumed to be moderately deleterious due to their lower translational accuracy [10, 11] and slower translation rate due to wobble-based decoding [12] and lower cognate tRNA levels [9, 13]. On the other hand, common codons were assumed to be preferred throughout selection events, leading to more efficient translation [14, 15]. However, translational kinetics that differ between synonymous codons play a vital role. For example, ribosomal pauses induced by translation rate variations offer additional time for correct cotranslational folding of certain protein domains or structural motifs, something that is supported by the enrichment of conserved rare codons in α-helices and adverse effects on protein synthesis upon substitution of these rare codons with common ones [9, 16]. To date, multiple studies have suggested that a dynamic translation rate is crucial for efficient protein biogenesis, indicating the need for low-frequency codons [3, 17, 18].

The growing awareness surrounding the importance of species-specific codon usage biases is reflected in the way gene design algorithms evolved to optimize protein expression [19]. The earlier codon optimization algorithms regarded rare codons as suboptimal and aimed to exchange them for more frequently used codons of the heterologous host [13, 20]. Initially, the ‘one amino acid-one codon’ strategy focused on replacing all synonymous codons with the host’s most prevalent codon under the presumption that charged tRNA molecules were not rate limiting [21–24]. However, significant growth inhibition is often observed due to imbalanced tRNA pools [25] and unwanted repetitive elements or secondary mRNA structures [13, 22]. The lack of flexibility in the ‘one amino acid-one codon’ algorithm and associated drawbacks resulted in the exploration of different algorithms, such as codon randomization [6, 26]. Although in some cases heterologous protein expression improved significantly when using these approaches [27, 28], the high translation rates still led to insoluble aggregates isolated in inclusion bodies [29] or to unsatisfactory expression in other instances [20, 30, 31]. While the influence of translation kinetics, originating from codon usage bias, on cellular processes such as chaperone interactions or cotranslational folding became apparent, new algorithms substituting native codons with synonymous ones while mimicking the original host’s pattern of codon frequency, i.e., codon harmonization algorithms, were explored and gained interest in ensuring the biogenesis of soluble, natively folded proteins [3, 32]. Nevertheless, as the synthetic biology community has not reached a consensus on whether codon harmonization (a strategy focused on quality and accurate translation kinetics) or codon optimization (a strategy focused on fast translation and protein quantity) is the superior strategy, recently developed tools support both codon harmonization and optimization and leave the choice to the user. While the main principle behind harmonization remains consistent, varying harmonization algorithms are employed by more recently developed proprietary and open-source software tools for synthetic gene design (Table 1). This fact, alongside several tool-specific sequence customization options, makes it hard to predict which tool offers the best codon harmonization for expression in a given heterologous system. Various studies concerning the drawbacks and strengths in different codon optimization tools have been carried out [19, 28, 33], but at the time of writing, comparisons of harmonization algorithms remained unexplored. Thus, to lay groundwork for future experimental research, we aim to examine the *in silico* performance of various tools in different model organisms. Hence, we have investigated four open-source genetic design tools that make use of codon harmonization algorithms and were available (EuGene, Galaxy, CodonWizard and CHARMING) [34–37]) to determine which of these tools, if any, is more suitable when redesigning certain genes or domains. CHARMING has two different modes of action, geometric mean (CHARMING:Geo) and %MinMax (CHARMING:MM); hence, a total of five tool outputs were evaluated.

The main reasons these four were selected, compared to the other tools mentioned in Table 1, were their public availability and target host range. Tools that required subscription or payment were excluded to ensure broad applicability within the scientific community. Additionally, tools that can only perform gene optimization or gene harmonization toward specific, predetermined heterologous hosts were not included. Genes were harmonized without selection of additional filters or options, as different tools possess different filters (Supplementary Table 1); thus, their effect on codon harmonization accuracy could not be accounted for. It is important to preface that the purpose of the intended research is to act as an exploratory study with the aim of evaluating different codon harmonization tools and the relevance of biological factors influencing the efficacy of harmonization results, thus paving the way for future studies and discussions regarding the topic.

Therefore, the four tools were selected for an *in silico* evaluation of their performance alongside an assessment of the influence on harmonization accuracy of various gene-specific characteristics (GC content, RNA folding and enzyme class) as well as of the choice for a heterologous host (*Escherichia coli, Saccharomyces cerevisiae* or *Streptomyces lividans*). To this end, generalized estimating equations (GEE) were used [38]. The selected gene characteristics were investigated due to their individual importance for gene expression and are expected to affect harmonization results. GC content has been correlated with gene expression levels [39], and organisms possess genomes with varying degrees of GC composition, indicating that this parameter might be important in codon harmonization. Aside from its implications for transcription and translation, GC composition has also been shown to strongly determine genome-wide codon bias, in turn influencing intergenetic codon rarities [40, 41]. RNA folding, on the other hand, is affected by synonymous mutations, as these are able to introduce new or eliminate existing mRNA secondary structures, which has an impact on mRNA stability and protein levels [42]. Last, enzyme classes can be predicted based on DNA sequence similarities and are expected to have specific but shared DNA motifs or domains [43–45]; hence, different classes are expected to behave differently in conjunction with harmonization efforts.

**Table 1:** Summary of codon harmonization/optimization tools and their characteristics

NAME	OPEN-SOURCE	INPUTS REQUIRED	FORMAT	CODON…	OTHER REMARKS	REFERENCE
GALAXY	Yes	- Genome natural & target host - Nucleotide sequence	Webpage	… harmonization		[35]
EUGENE	Yes	- Genome natural & target host - Nucleotide sequence	Software	… optimization & harmonization	- Several gene redesign options - Time consuming	[34]
CODON- WIZARD	Yes	- Genome natural & target host - Codon usage table (CUT) - Nucleotide sequence	Software	… optimization & harmonization	- Several gene redesign options	[36]
CHARMING	Yes	- CUT natural & target host - Nucleotide sequence - Codon usage measure - Window size	Webpage/Software	… harmonization	- Downloadable Python code - Equally well harmonized synonymous outputs possible	[37]
CAD4BIO BGene	No	- Company contact information	Webpage	… optimization & harmonization		[31]
GeneWiz by Azenta	No	- Company contact information	Webpage	… optimization		[46]
Atum.bio DNA2.0 Gene Designer	No	- Company contact information	Webpage	… optimization		[22]
GeneArt GeneOptimizer Thermofisher	Yes	- Target host - Nucleotide sequence	Webpage	… optimization		[47]

## Results

**Fig. 1:**
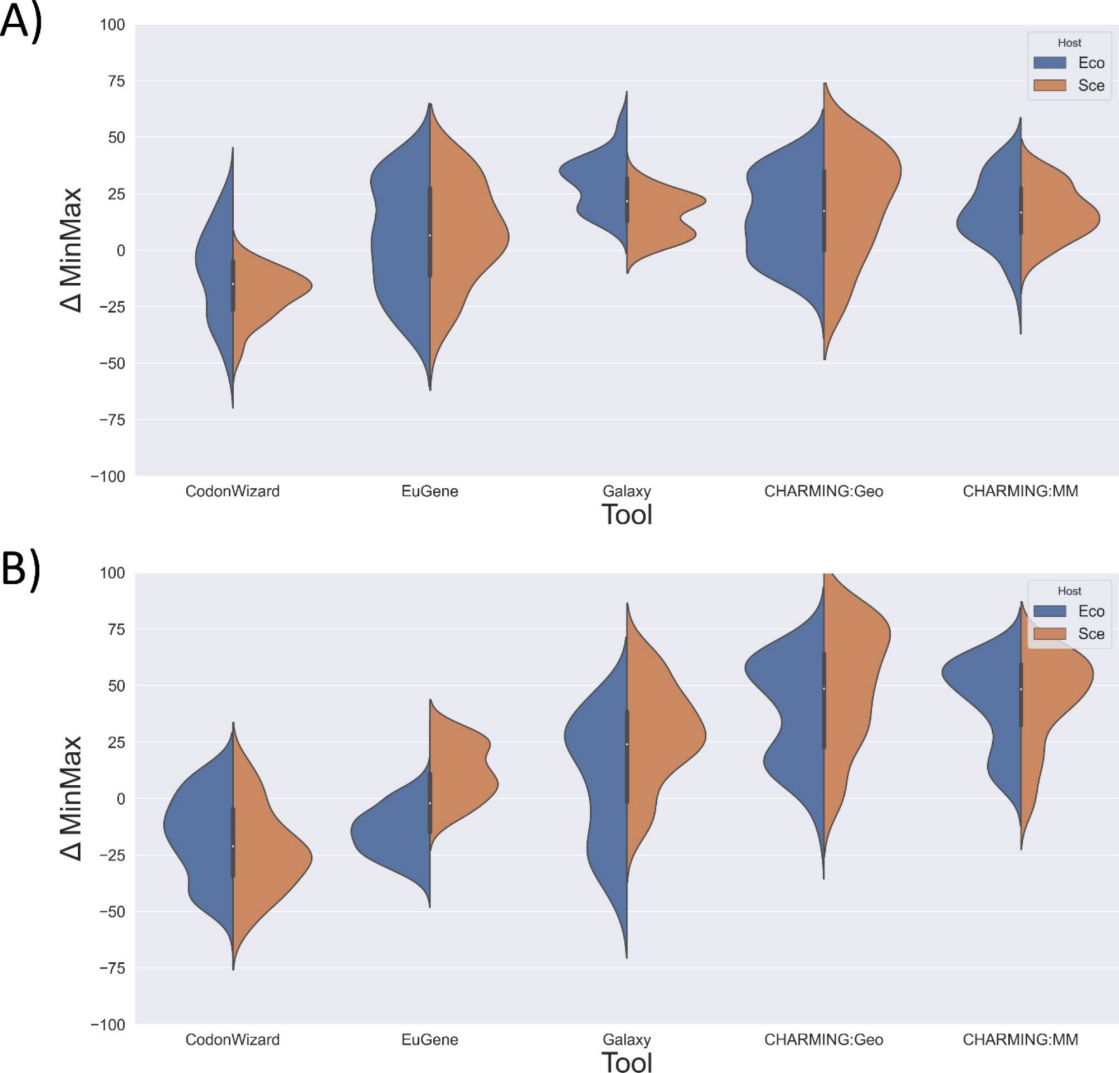
Violin plots of the genes (**A**) NMB0255 and (**B**) VGB. In these plots, ΔMinMax is displayed as a distribution on the vertical axis for each of the tool-host combinations (Escherichia coli = Eco and Saccharomyces cerevisiae = Sce). The ΔMinMax values should be closely distributed around zero. Both modes of CHARMING, geometric mean (Geo) and %MinMax (MM), were evaluated

With each of the investigated codon harmonization tools aiming to nullify the codon usage bias between the original and the intended host, one would expect similar results across tools. This was investigated by calculating the relative codon usage frequencies (%MinMax) for all 27 genes from the genetic dataset, harmonized to *E. coli* (Eco) or *S. cerevisiae* (Sce), and comparing them to the original distribution (ΔMinMax), visualized by violin plots (Supplementary Fig. 1). As an example, the violin plots of 2 genes are shown in Fig. 1. The distribution of the difference in codon usage frequency between the original and the heterologous host (Y-axis) should center around zero. The farther away from zero, the less optimal the harmonization tool performed. Clear differences in the distributions between both tools and hosts (*Escherichia coli* and *Saccharomyces cerevisiae*) were observed, meaning that certain tools perform better in their codon harmonization tasks than others. An important note to make is that CodonWizard and CHARMING use the outdated Kazusa database for information on CUTs, while the others employ the more frequently updated HIVE-CUT database. To account for this discrepancy, the correct CUTs were factored in when analyzing the codon harmonization tools, ensuring that the tools could be compared to one another.

**Fig. 2:**
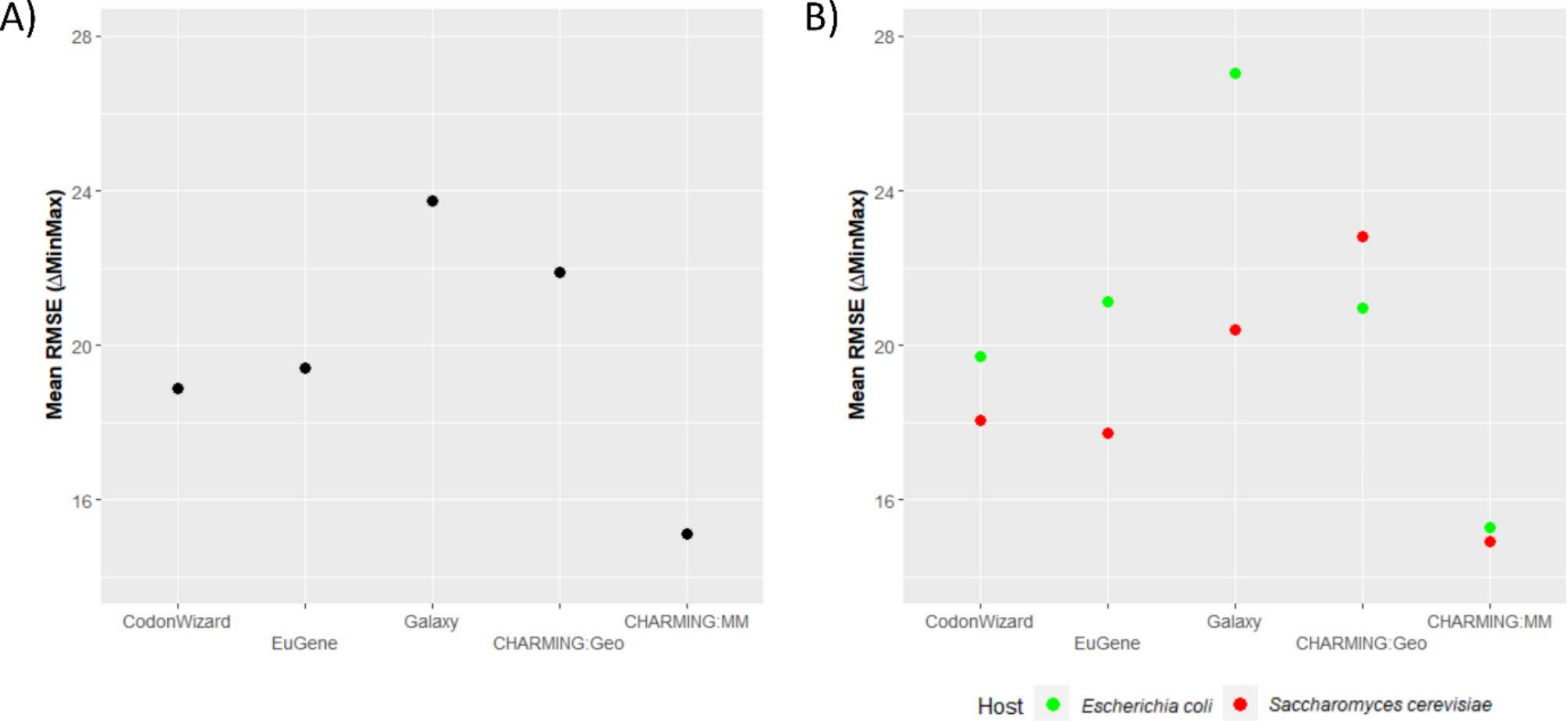
In **A**), the mean RMSE is plotted for each tool, while in **B**), the effect of the host on the mean RMSE is plotted for each tool. A lower mean RMSE indicates a better performing codon harmonization tool

To further investigate the difference in performance between the codon harmonization tools, the influence of various gene-specific characteristics (GC content, RNA folding and enzyme class) as well as of the heterologous host on the harmonization accuracy was assessed for each of the tools. First, a general comparison was made in which the tools were compared to each other as a whole. To do this, per gene, the root mean square error (RMSE) was derived from the ΔMinMax values calculated between the original and the harmonized gene (see Materials and Methods section). The mean RMSE values (mean of all 27 genes under investigation) of each tool are plotted in Fig. 2A, confirming clear differences in tool efficacy. To enable a statistically substantiated evaluation of the different tools and the influence of the different parameters on their codon harmonization efficiency, GEEs were fitted, whereafter the Wald test, commonly used to calculate p values and confidence intervals of GEEs, was performed. Again, from the Wald test between the equation with and without the tool-incorporated factor, it was clear that the tools have a significant impact on the mean RMSE (p value of 2.0 *10^− 16^), indicating that, in general, the tools could be organized from the lowest mean RMSE to the highest, i.e., from the best performing to the worst performing as follows: CHARMING:MM, CodonWizard, EuGene, CHARMING:Geo & Galaxy.

Next, it was examined whether the choice for a given heterologous host would affect the codon harmonization efficiency of the tools. Hereto, the model organisms *E. coli* and baker’s yeast were selected. In Fig. 2B, the mean RMSE was plotted as a function of each tool, calculated separately for each heterologous host. While clear differences between tools are still visible, codon harmonization toward *E. coli* generally yields a higher mean RMSE than toward *S. cerevisiae*, with the exception of CHARMING:Geo. Similar to before, the CHARMING tool in MM mode scores very well for both hosts, whereas the difference for Galaxy is the largest. These findings were also supported after statistical analysis, as the choice of host had a significant effect (p value of 2.9 *10^− 3^) on the mean RMSE values, and this effect was dependent on which tool was used for codon harmonization, as the interaction term was significant as well (p value of 1.2 *10^− 3^).

**Fig. 3:**
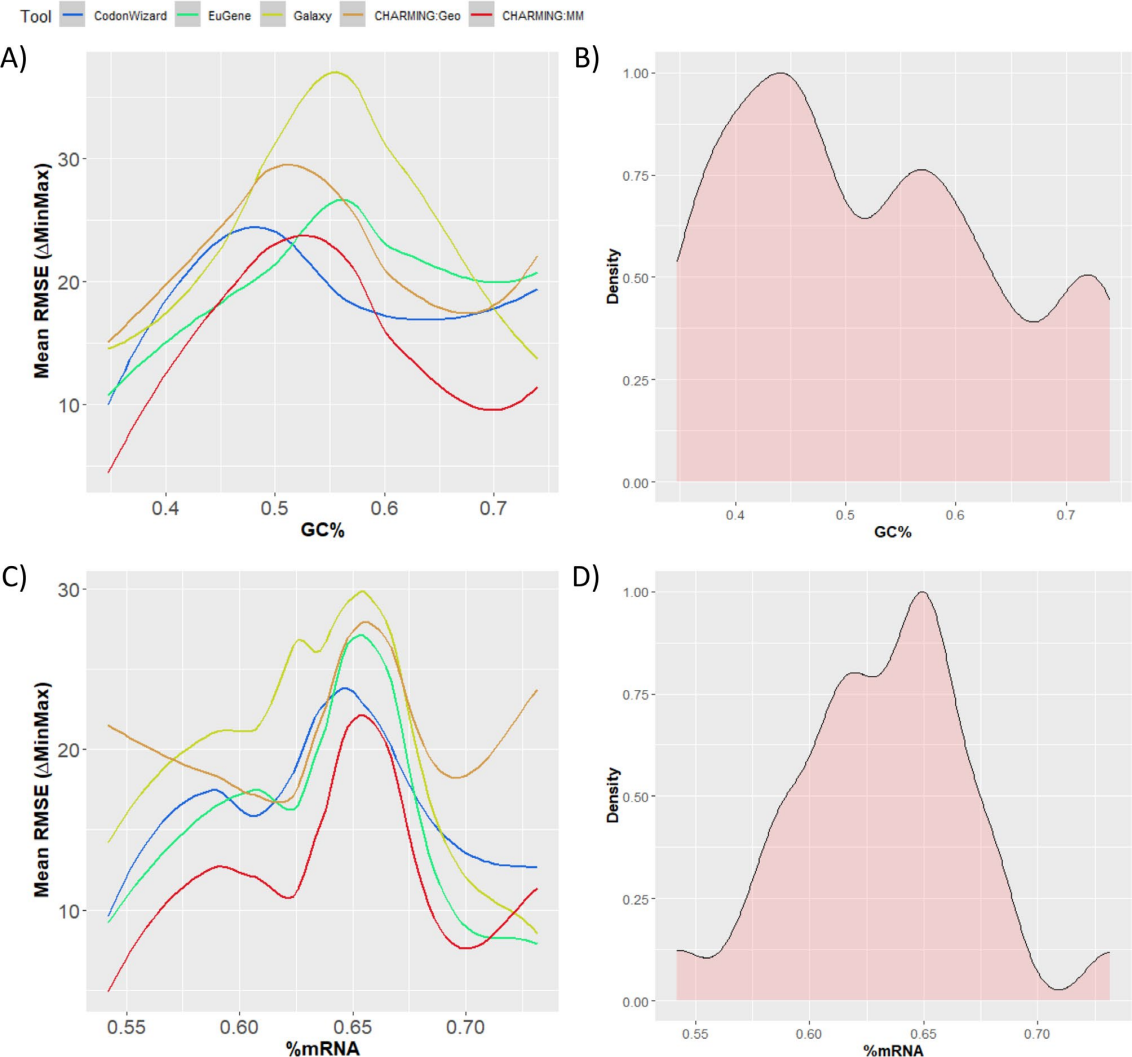
Plot **A**) shows the effect of GC content on the mean RMSE and thus the harmonization results. In **B**), the distribution of GC content in the data is displayed. **C**) plots the effect of secondary structures (%mRNA) on mean RMSE, while D) displays the distribution of the %mRNA in the dataset

As the GC content of a gene plays an important role in protein formation and gene expression [48, 49], its effect on codon harmonization was also investigated. Figure 3A and 3B therefore represent the mean RMSE as a function of the GC content and the distribution of the GC content within the gene dataset, respectively. From Fig. 3B, it was clear that there is no preference for a certain GC range, and all %GC values are more or less evenly represented, between a content of 30% and 80%. For every tool, the GC content affects the mean RMSE and hence the harmonization accuracy. In general, for all the tools, the highest mean RMSE values (and thus worst results) were obtained in the 45–60% GC range. The mean RMSE values gradually decrease for lower or higher GC contents. Overall, CodonWizard (maximal difference in mean RMSE of 9) and CHARMING:Geo and EuGene (maximal difference in mean RMSE of 15) seem to be the least influenced by the GC content of a gene, while Galaxy and CHARMING:MM are the most influenced (maximal difference in mean RMSE of 19 and 22, respectively). However, CHARMING:MM has in general the lowest mean RMSE, indicating that it performs most accurately over all. The parameter ‘GC-content’ and its interaction term were added to the equation, and the Wald test was used to investigate their effect on the parameter Tool. The resulting p value is 1.9 *10^− 7^, meaning that GC content indeed has a significant impact on the mean RMSE and thus on the codon harmonization results. Afterwards, it was checked if the interaction term between Tool and GC-content is necessary. The comparison between both equations rendered a p value of 2.15 *10^− 15^, meaning that the effect of GC content on the results is dependent on the choice of tool.

mRNA secondary structures formed by, e.g., intramolecular interactions, can also play an important role in controlling translation [50], warranting closer inspection pertaining to codon harmonization tools. In Fig. 3C, the mean RMSE was plotted as a function of the amount of secondary structures present in the original gene sequence, indicated by %mRNA. %mRNA is the percentage of mRNA of a sequence affected by secondary structures. This value was calculated using RNAfold with default settings. As expected, mRNA secondary structures have a clear effect on codon harmonization efficiency, with a similar pattern for each tool, reaching a maximum mean RMSE of approximately 0.65% mRNA. However, the magnitude of the effect is dependent on the tool. CHARMING:Geo is the least affected and performs the most consistently, along with CodonWizard. Galaxy, on the other hand, is once again the most influenced by biological parameters. As for the GC content, CHARMING:MM has in general the lowest mean RMSE and hence performs most accurately over all %mRNA values, in contrast to CHARMING:Geo. After performing a Wald test, it was proven that the parameter %mRNA has a significant effect on the mean RMSE and thus the harmonization results (p value of 6.61 *10^− 11^). No statistical analyses have been performed on the interaction term due to the size of the genetic dataset: not enough observations are available to prevent overfitting when estimating the needed model parameters. Figure 3D shows the distribution of the mean %mRNA of the various genes. There are relatively more datapoints with mean %mRNA values between 0.60 and 0.67. Overall, a normal distribution was obtained for %mRNA values between 0.58 and 0.68.

**Fig. 4:**
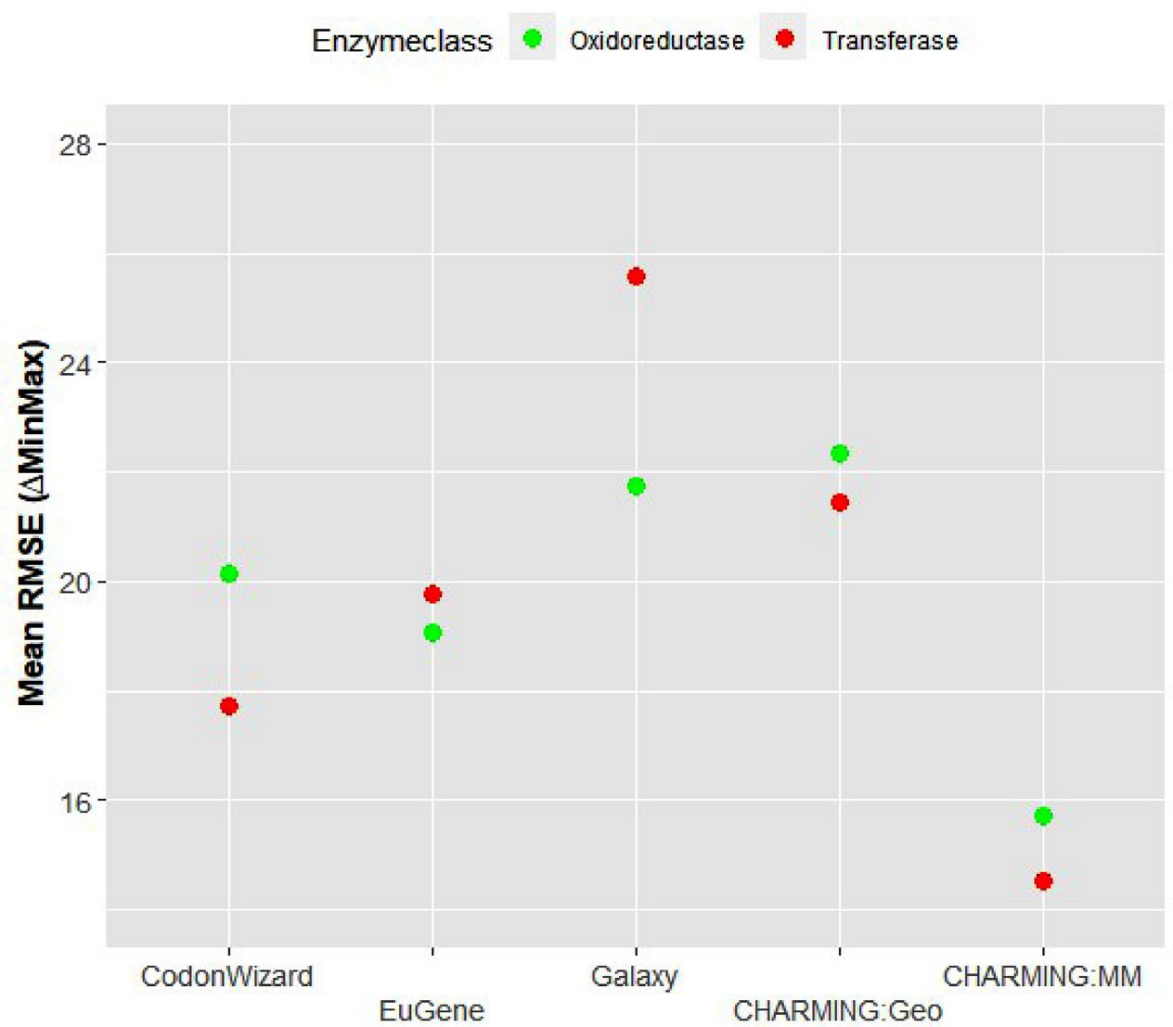
The effect of enzyme class on the mean RMSE and how this is possibly affected by the choice of tool. Green represents the oxidoreductase results, red the transferase results

The last biological parameter that was taken into account is the enzyme class to which the gene product belongs. The enzyme class could potentially have an effect on codon harmonization, as this is often accompanied by the occurrence of different DNA motifs or domains, such as transmembrane regions, metal binding and repeating regions. Here, the enzyme class was plotted versus the mean RMSE, showing that the differences between both enzyme classes are less pronounced (Fig. 4). After the Wald test of this parameter both with and without the interaction term (p values of 0.90 and 0.85, respectively), it could be concluded that it has no effect on RMSE and thus on codon harmonization.

**Fig. 5:**
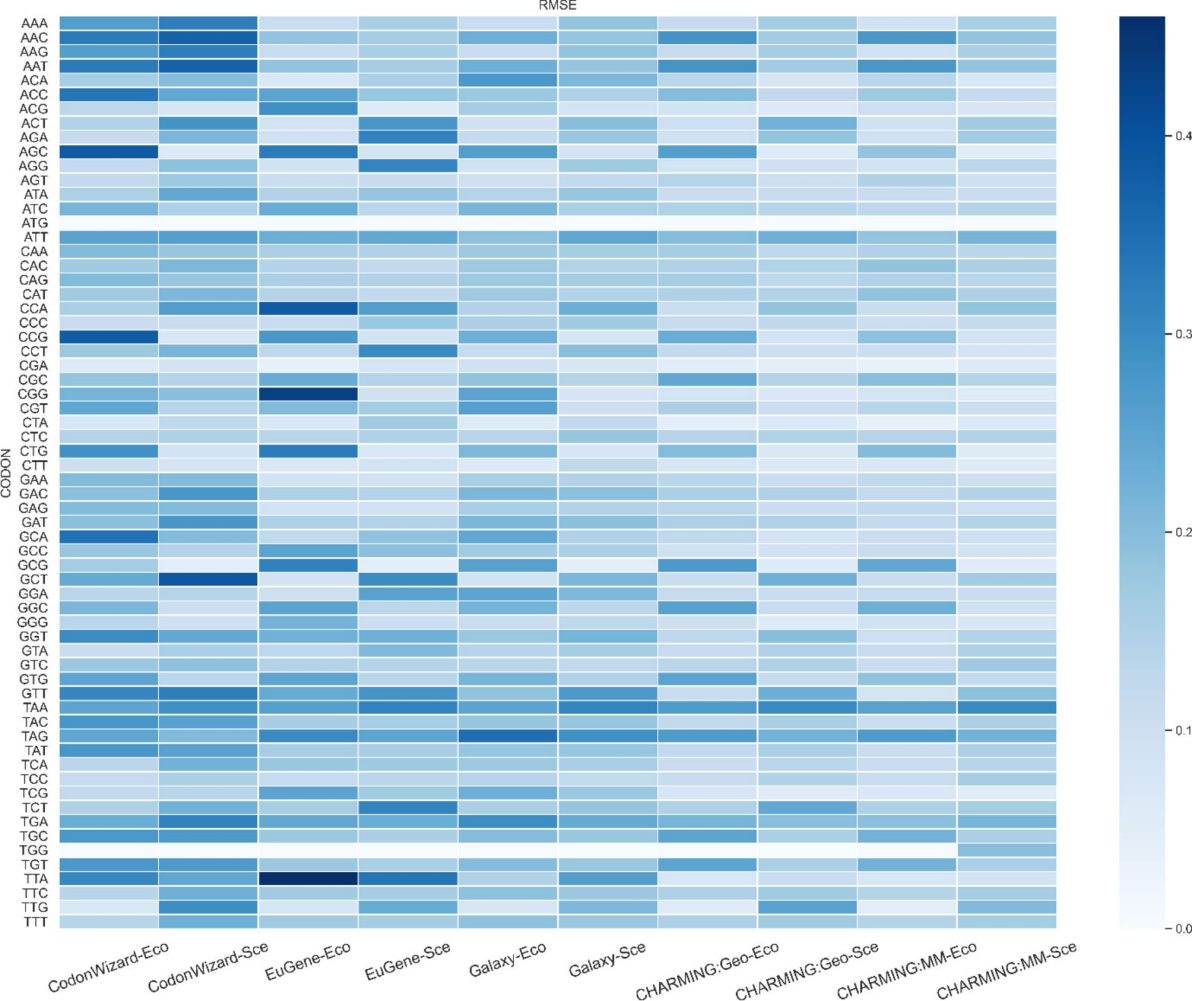
Codon change heatmap displaying separately calculated RMSE _*codons*_ for each codon vs. tool-host combination. As some codons do not have synonymous codons (ATG: M, TGG: W), they yield an RMSE of 0

In addition to analyzing the codon harmonization tools in a general manner or the effect of various biological parameters on their performance, a closer investigation as to whether each codon is equally efficiently harmonized by each tool was conducted. To do so, heatmaps visualizing the RMSE_codons_ for each tool were made. This RMSE_codons_ (see Methods section) is a measure for the CUT-corrected difference in occurrence of a certain codon between all 27 original sequences and harmonized sequences, or, put otherwise, it is a measure for which codons are harder to harmonize than others. The better the tool could handle the harmonization, the closer to zero its RMSE_codons_ should be, and thus, when visualized as a gradient of blue in a heatmap, the whiter it should appear (Fig. 5). Figure 5 represents the RMSE_codons_ for all 64 unique codons for the 5 tool outputs and the 2 heterologous hosts.

The brighter areas of the heatmap are mostly situated on the right, while the darker blue cells are situated on the left. This again is an indication that CHARMING did a better job harmonizing the various genes from the dataset than the other tools. This finding is supported by Table 2, where the mean RMSE_codons_ values are presented, calculated over all different codons for each tool-host combination. Per specific codon, CodonWizard generally produced the highest RMSE values, while CHARMING produced the lowest RMSE values.

**Table 2:** Mean RMSE_codons_ for every tool-host combination

TOOL-HOST	MRMSE_codons_
CodonWizard *E. coli*	0.20
CodonWizard *S. cerevisiae*	0.20
EuGene *E. coli*	0.18
EuGene *S. cerevisiae*	0.16
Galaxy *E. coli*	0.17
Galaxy *S. cerevisiae*	0.16
CHARMING:Geo *E. coli*	0.14
CHARMING:Geo *S. cerevisiae*	0.13
CHARMING:MM *E. coli*	0.13
CHARMING:MM *S. cerevisiae*	0.13

To validate the findings of the sections above and as an example of how this research could be useful for future research, four case studies were worked out with academic and/or industrially relevant genes. To this end, a dataset with new genes was compiled, namely the validation dataset (Supplementary Table 3). The 4 case studies are:

1. Which tool is most reliable to harmonize a gene of interest having a high/low GC content?
2. Which tool is most reliable to harmonize a gene of interest that has/does not have secondary mRNA structure(s)?
3. Which tool is most reliable to harmonize a gene for expression in a model host like *E. coli* or *S. cerevisiae*?
4. Which tool is most reliable to harmonize a gene for expression in a non-conventional host like *Streptomyces lividans*?

**Fig. 6:**
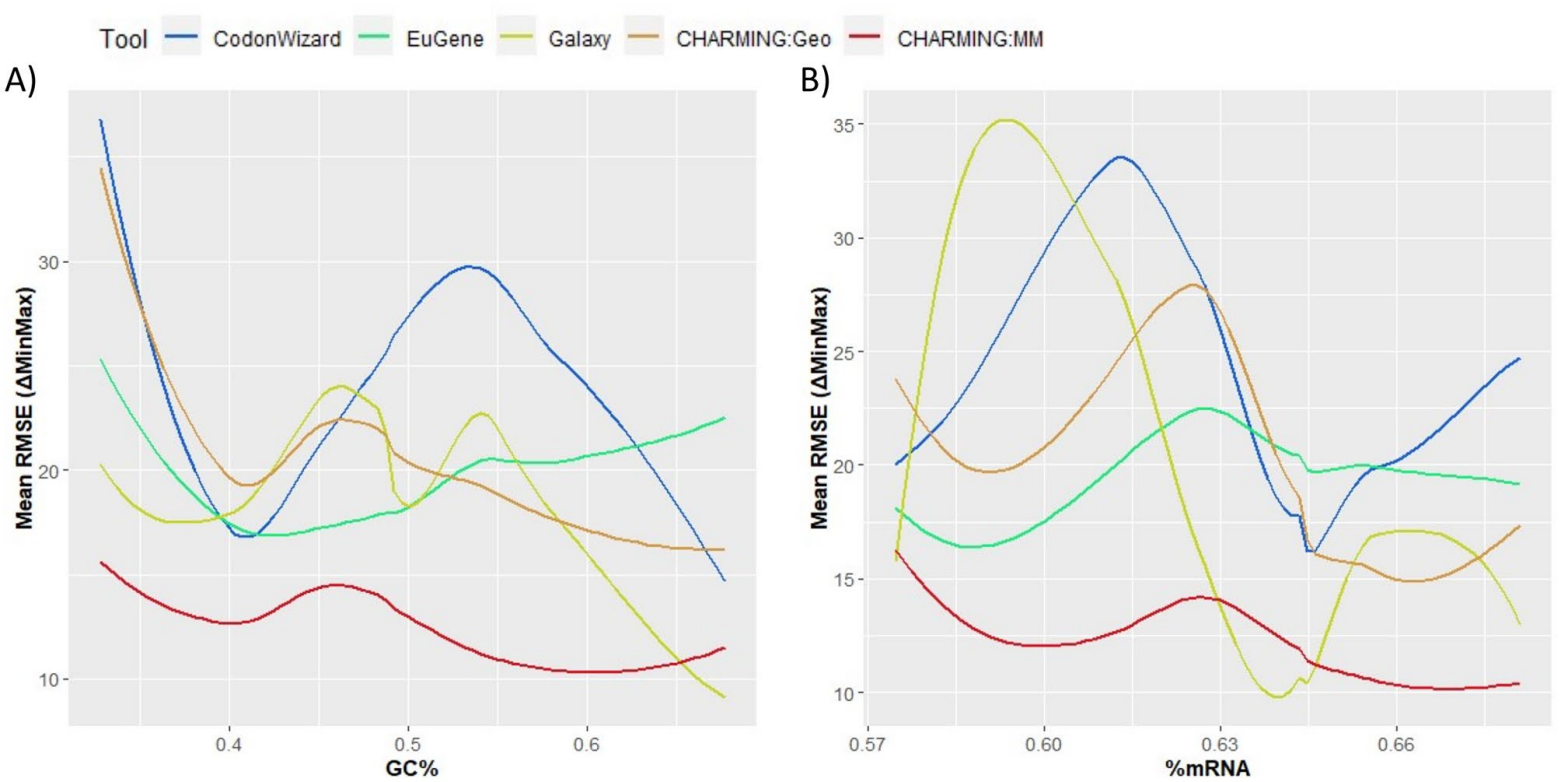
Plot **A**) shows the effect of GC content on the mean RMSE and thus the harmonization results for the validation dataset. In **B**), the effect of secondary structures (%mRNA) on mean RMSE is displayed

As visualized in Fig. 6A, analysis with the validation dataset confirmed the effect of GC content on the mean RMSE values, and thus on harmonization results, and confirmed a significantly different effect of GC content on harmonization results when using different tools. This was backed by statistical analysis, because a p-value lower than 2*10^− 16^ was observed for both the effect of GC content itself as its interaction term with tools. It was confirmed that CHARMING:MM performs the most robust over all GC content ranges evaluated (lowest mean RMSE) and hence can be chosen for harmonization purposes, regardless if your gene of interest has a high or a low GC content. Using the validation dataset, the extent to which harmonization is affected by GC content using the different tools was different as compared to the genetic dataset.

As for GC content, analysis with the validation dataset confirmed the effect of the prevalence of mRNA secondary structures on mean RMSE and seems to confirm the fact that it is different for different tools (Fig. 6B). The first finding was backed by statistical analysis, since a p-value of lower than 2*10^− 16^ was the outcome of the performed Wald test. The interaction term between %mRNA and tool could not be statistically identified as significant, since it could not be calculated, due to the complexity of the relation and the need for more data to reduce overfitting. Again, CHARMING:MM performs the most robust over all %mRNA ranges (lowest mean RMSE) and hence can be chosen for harmonization purposes, regardless if your gene of interest has or does not have secondary mRNA structure(s).

**Fig. 7:**
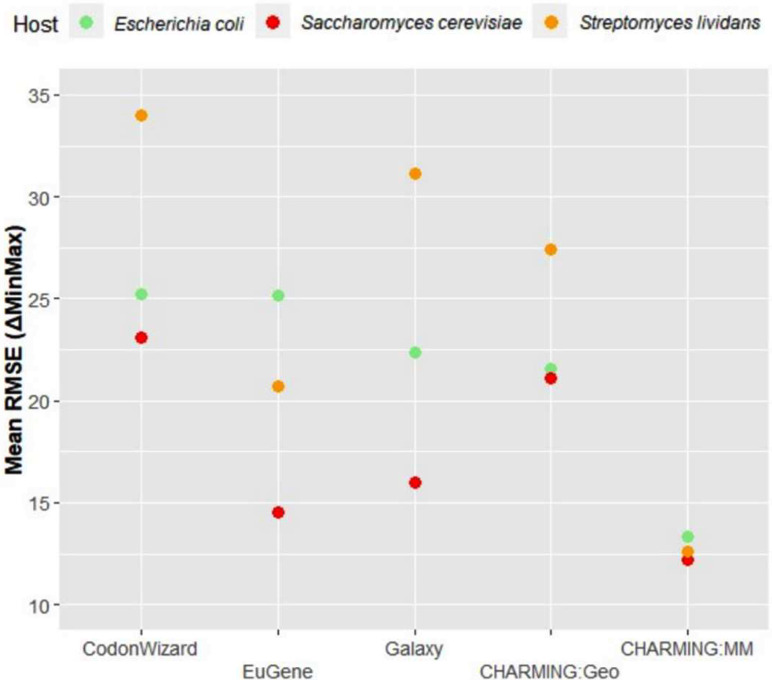
The effect of the choice of host on the mean RMSE is plotted for each tool, results obtained with the validation dataset. A lower mean RMSE indicates a better performing codon harmonization tool

For the third case study, we wanted to check which tool is the better choice to harmonize a gene for expression in a model host like *E. coli* or *S. cerevisiae.* In Fig. 7, the mean RMSE values for each tool-host combination obtained with the validation dataset are plotted. Clear differences can be seen between the combinations. The choice of host had a significant effect on mean RMSE values (0.15*10^− 3^) and the interaction term between host and tool was also significant (0.26*10^− 2^), meaning that the effect of host was dependent on the choice of tool. Overall, it was seen that harmonization towards *S. cerevisiae* resulted in lower mean RMSE values than harmonizing towards *E. coli*. Both CHARMING modes (Geo and MM) performed the most consistent for both production hosts, since the difference in mean RMSE values between the 2 hosts is the smallest for this tool. Contrary to the results obtained with the genetic dataset, using the validation dataset, EuGene performs the least consistent, having the biggest difference in mean RMSE values between *E. coli* and *S. cerevisiae*. However, the validation dataset is smaller than the genetic dataset. Regardless for which model host you harmonize your gene, the lowest mean RMSE values are obtained with CHARMING:MM, which confirms the results obtained with the genetic dataset.

**Fig. 8:**
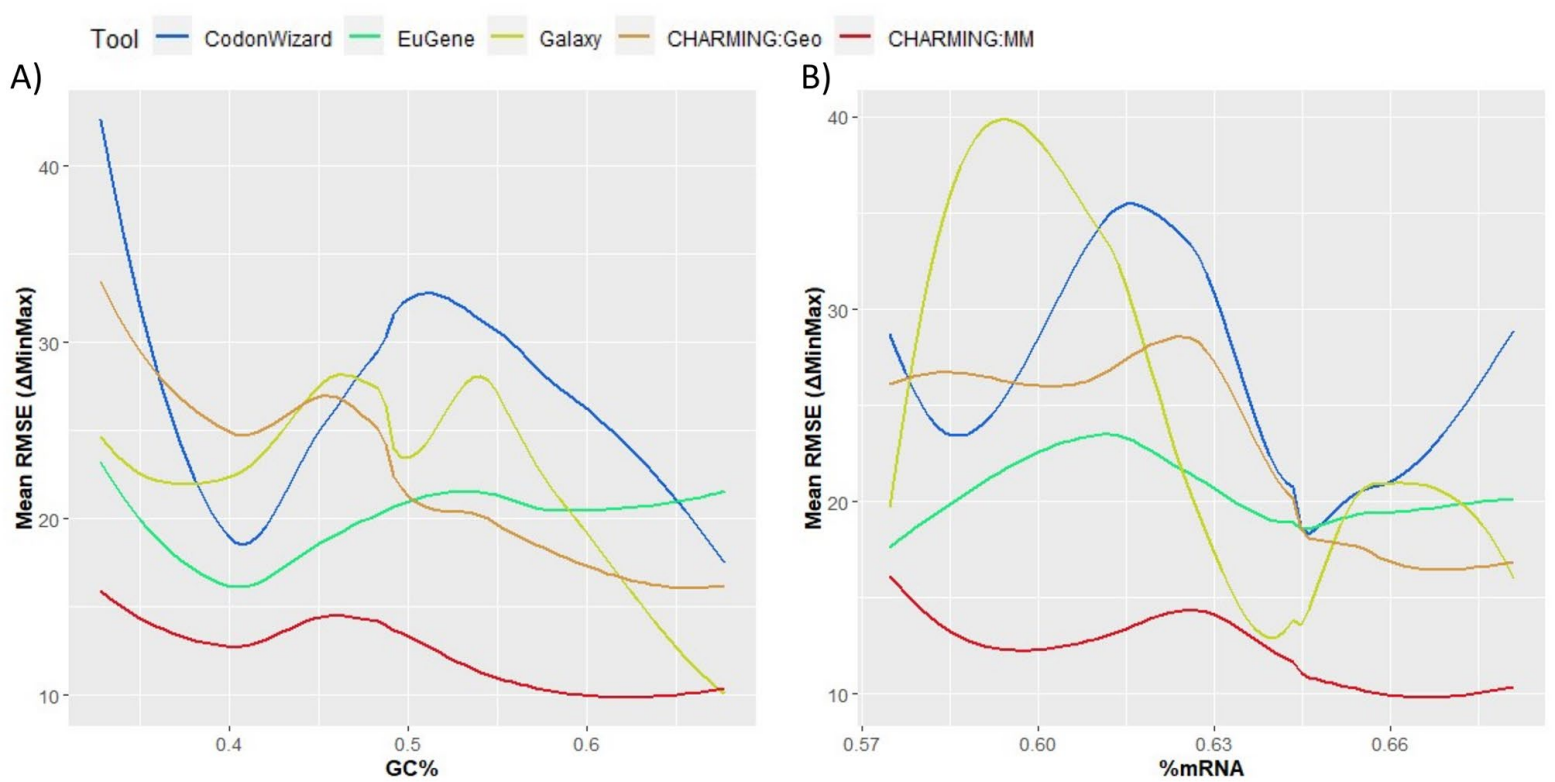
Plot (**A**) shows the relationship between GC% and mean RMSE values in the validation dataset for S. lividans, while plot (**B**) shows the effect of %mRNA on mean RMSE values in the validation dataset for S. lividans

As a fourth and final case study, the question of which tool to use when harmonizing a gene for expression in a non-conventional host was raised. Indeed, not all heterologous expression is performed in *S. cerevisiae* or *E. coli*, a plethora of other production hosts has been described in literature. Here, we considered *Streptomyces lividans* to harmonize the genes of the validation dataset towards. This microorganism has already been well characterized and is known for its high genetic tractability. It is often used in literature for the production of secondary metabolites, so codon harmonization towards it is of importance. From Fig. 7, it can be seen that harmonization towards *S. lividans* generally results in higher mean RMSE values and thus worse harmonization results. Here again, CHARMING:MM is the most consistent harmonization tool both delivering the lowest mean RMSE results as the lowest difference in mean RMSE results between various hosts. It can hence be advised for use when harmonizing genes for *S. lividans.* Since for different tools harmonization towards *S. lividans* was much worse than for model organisms like *E. coli* and *S. cerevisiae*, the other biological parameters were taken under the loop as well (Fig. 8). When comparing both for GC content and %mRNA the mean RMSE graphs obtained with the validations set for the reference organisms (Fig. 6A and B, respectively) with those obtained for *S. lividans* (Fig. 8A and B, respectively), strong resemblances between them can be seen and the same conclusions can be drawn: also for harmonizing a gene for use in *S. lividans*, regardless whether the original gene has a high or low GC content or weak or strong mRNA structures, using CHARMING:MM will result in the lowest RMSE values. After performing the Wald test, the effect of GC content on mean RMSE values remains significant and the interaction term between GC content and tool does so as well (both p-values are lower than 2*10^− 16^). Likewise, after erforming the Wald test, the effect of mRNA is still significant (p-value lower than 2*10^− 16^).

## Discussion

It can be argued that one of the motivators for the continued development of new harmonization tools is the lack of consensus on which codon usage measure is to be employed in harmonization efforts [51]. Here, we considered using both the codon adaptation index (CAI) and %MinMax for the comparison of the open-source harmonization tools. CAI is one of the most commonly used and earliest codon usage measures [52], utilized by many commercial vendors [53], while the patterns calculated by the %MinMax algorithm are predictive of the translational kinetics of nascent polypeptide chains [54] and have helped steer the protein folding mechanism in an expected manner [55]. Despite its popularity in synthetic gene design, concerns about CAI being poorly predictive of protein yield are becoming increasingly valid, as reports have pointed out that there is no correlation between protein expression levels and CAI [42, 56], which has been reaffirmed in a recent study [33], leading to our choice of %MinMax as the more reliable codon usage measure for tool comparison.

Turning the data into violin plots, differences in output between codon harmonization tools became clear. To shed light on why these tools differ and how they can cope with various biological parameters, an in-depth analysis was performed to help select the most suitable tool for codon harmonization. Based on the Wald test, p values were delivered that showed that the codon harmonization efficiency differs significantly with regard to the tools and hosts. According to these findings, the tools could generally be ranked, from better performing to worse, as follows: CHARMING:MM > CodonWizard > EuGene > CHARMING:Geo > Galaxy. When hosts are taken into account, the order changes as follows: CHARMING:MM > EuGene ≈ CodonWizard > Galaxy > CHARMING:Geo for *Saccharomyces*, CHARMING:MM > CodonWizard > EuGene ≈ CHARMING:Geo > Galaxy for *E. coli.* Looking at the biological parameters GC-content and %mRNA, a more complex effect on harmonization results was observed. It could be seen that both GC content and RNA folding have the biggest effect on RMSE values, and thus harmonization results, of Galaxy, leading again to the indication that this tool might be the least suitable. Contrary to previous results, CodonWizard instead of CHARMING:MM seems to be the least influenced by variations in GC content or RNA folding. Despite the correlation of DNA motifs or regions such as transmembrane regions, metal binding and repeating regions with certain enzyme classes, no significant effect of enzyme class on codon harmonization results was observed. Finally, it became apparent that the open-source tools investigated in this manuscript also differ at the codon level. CHARMING and Galaxy are more capable of resembling the original codon usage frequency in the harmonized genes for both *E. coli* and *S. cerevisiae*. Additionally, for certain codons, it is easier to nullify the difference in occurrence between the original and harmonized genes, and this effect was different for the various tools.

To verify our conclusions in regards to the tools’ harmonization efficacy, a validation data set was used for four different case studies, as described in the Results section. Genes for this dataset were selected in a manner that ensured enough variability for each of the investigated biological factors. As with the original dataset, the influence of GC-content, %mRNA and choice of host is again shown to significantly influence harmonization results of each tool, substantiating our previous findings. More importantly, the results further confirm that CHARMING:MM can be considered as the most efficient and most reliable tool for giving more consistent harmonization results, as it shows the lowest mean RMSE over both varying GC-content and %mRNA. Similarly, it remains consistent in its harmonization results with the choice of *S. lividans* as host, as opposed to CHARMING:Geo and CodonWizard, which show a stark departure from the previous consistent results in regards to the choice of host, as a large deviation from the mean RMSE when harmonizing towards *E. coli* and *S. cerevisiae* is observed. The results suggest that CHARMING:MM seems to be the most robust choice for harmonizing towards these three heterologous hosts.

As mentioned at the start of the Results section, harmonization with CodonWizard and CHARMING is standardly performed by making use of the CUTs on the Kazusa database, which has been outdated since 2007. The scarce number of coding sequences for many organisms listed in this database is a pressing issue. The other tools allow for employing a user-specified CUT in high throughput, which, for example, can be derived from the newer, regularly updated HIVE-CUT database, which hosts codon usage statistics for every organism that has available sequencing data on RefSeq or GenBank [57], thus increasing available codon usage statistics both in size and accuracy. As harmonization heavily depends on the accuracy of the CUTs, tools that are able to incorporate HIVE-CUT data are invaluable in the future. CodonWizard aims to adopt this functionality in the future [36]. When the user wants to use their own CUT, every codon has to be imported separately, making it extremely cumbersome when harmonizing genes from various organisms. Galaxy is also able to calculate CUTs from user-uploaded genomes and generates downloadable CSV files that can also be edited, creating a more transparent process. In addition to the input, each of the tools differs in the algorithm used for codon harmonization. In the case of EuGene, harmonization can be performed with either of two codon usage measures: relative synonymous codon usage (RSCU) or CAI [52]. The algorithm scans each codon one by one, considering all synonymous codons and their usage in the host species, and selects the one with a minimal difference in RSCU or CAI, depending on the user’s choice, to that of the original species (Paulo Gaspar, personal communication, 15/02/2021). Galaxy, on the other hand, converts the CUT to relative codon adaptiveness scores, a measure based on the RSCU values originally devised by Sharp et al. that represents the frequency of a codon compared to the frequency of the most prevalent synonymous codon. Whereas codon usage bias is regularly determined based on a reference set of highly expressed genes of the organism [4, 6], scores calculated by Galaxy are based on the codons of all protein-encoding genes of a genome assembly, i.e., on the complete ORFeome [35, 52]. Using these scores for comparison, the harmonization algorithm finds the best matching synonymous codons for the target gene in the new expression environment. CodonWizard employs a variety of algorithms and allows for less stringent codon modification criteria through the concept of ‘tolerance’. It offers an empirical approach to optimize heterologous protein expression and to accommodate for the lack of clear understanding of all complex aspects influencing translation efficiency. The harmonization algorithm calculates the absolute difference of each synonymous codon’s relative usage frequency in the heterologous host with that of the original host’s codon. These differences are then converted to probabilities that dictate the likelihood that a codon is selected for replacing the original codon. Codons with a smaller difference from the original codon have a higher likelihood of being selected. The basic harmonization algorithm will select the codon with the lowest absolute difference as a replacement. However, by introducing a specific tolerance level, a pool of potential candidates formed from the synonymous codons, which is based on the calculated probabilities, will be considered for replacing the original codon. The tolerance level determines the size of this pool, ranging from zero tolerance featuring solely the most similarly frequent codon to consideration of every synonymous codon at 100% tolerance. To avoid confounding, codons are analyzed and adapted in a random manner instead of sequentially in a 5’-to-3’ direction (Peter Rehbein, personal communication, 15/05/2021). Finally, CHARMING stands for Codon HARMonizING and is an upgrade of the rudimentary codon harmonization algorithm ‘Rodriguez initialization’, which was previously developed alongside a tool for evaluating codon usage patterns [37, 54]. The synonymous codon sequence is analyzed based on the codon usage values of the destination host and a user-specified sliding window, assigning values to each individual codon. A comparison to the wild-type values of the original host serves to identify potential codon alterations. When a local optimum has been achieved and the algorithm is unable to decrease the net deviation any further, the output is final. Since a given input will always return the same output, a possibility to explore other local optima is offered through the option of generating random synonymous sequences that are subsequently used as alternative inputs and in turn produce equally well harmonized but unique solutions.

Another important factor to take into account when comparing the various tools is the user-friendliness and presence of customization through various filters (e.g., site removal, amino acid starvation, secondary structure of RNA optimization), which also greatly varied between the tools. In general, EuGene is the most flexible tool with various features allowing gene tailoring (see Supplementary Table 1), while CHARMING and Galaxy possess none. Importantly, when additional filters are used in EuGene, the rendering time greatly increases, making it very slow. Additionally, the results obtained differ greatly between uploading the heterologous host first or selecting the host in the drop-down menu. The redesign criteria either support a simulated annealing approach to rapidly approximate a global optimum or the calculation of several Pareto-optimal solutions using a genetic algorithm from which the user can choose. To automatically prefer harmonization solutions over other design criteria, an option to favor retaining rare codons is present. This prevents the program from removing a rare codon despite the event of its substitution for a more frequent codon drastically increasing the quality with respect to a different selected design criterion (e.g., codon context), although it is unclear at what frequency the algorithm considers a codon as ‘rare’. Another useful feature is the gene diagnosis option, which scans the selected gene and returns information related to any of the selected redesigns. This significantly facilitates further examination of redesigned or original genes, as well as methods and allows comparisons between them. However, due to scarce documentation combined with the use of percentages to indicate levels of improvement instead of established scores, effective interpretation of the exact improvements remains complicated [19]. CodonWizard has some filters, but with a rather limited use (e.g., amino acid starvation only for *E. coli*). After finishing harmonization, a report is generated featuring relevant diagnostics such as GC%, codons changed and a graphical representation of codon usages, albeit lacking X- and Y-axis variables, obfuscating interpretation. Finally, CHARMING’s web application is limited to only 350 codons, making the harmonization of large genes cumbersome. To do so, the user needs to download the original python file of the tool and harmonize its gene there. In general, the rendering time of CHARMING can be long for large genes as well. In this paper, CHARMING was also tested by using the Kazusa database. This was because CHARMING was better adapted for the use of Kazusa and could be used more efficiently with this database in a high-throughput context. However, it could also be used with other CUT databases.

## Conclusions

As protein expression of genes in heterologous hosts is of vital importance to metabolic engineering and protein production to establish microbial cell factories, the purpose of this paper is to shed light on the capabilities of currently available open-source codon harmonization tools. The current genetic dataset was too limited to allow for highly complex models to be made without significant loss of statistical power, yet it provides a foundation for future research and comparison of these tools through such models. While the effect of various parameters, such as heterologous host, GC content and secondary structures, was clearly observed, the enzyme class did not significantly impact codon harmonization results. Despite the lack of statistical power to investigate these parameters altogether, CHARMING with the %MinMax mode enabled seems to be the most promising tool for most gene designs, regardless the choice for the heterologous host, the gene of interests’ GC content or the prevalence of mRNA structures. However, when the user intends to further customize their genetic code, perhaps tools such as EuGene and CodonWizard could be opted for in a second round of harmonization, after using CHARMING:MM, as they offer additional filters for gene tailoring and optimization, meaning automated custom design for e.g. removal of preliminary transcription termination signals, restriction enzyme recognition sites, mRNA secondary structures etc. Our findings also lead to the belief that Galaxy is the least performing codon harmonization tool. Attention should also be given to the input required by the tools. CodonWizard and CHARMING employ, in a standard setting, the outdated Kazusa CUTs, while other tools allow for user-specific inputs from, for example, the HIVE-CUT database. In addition to the effect of biological parameters, differences between tools were also observed at the codon level, where certain tool-host combinations were more capable of resembling the native codon usage frequency. Although various parameters seem to have a significant effect on codon harmonization, the impact of these substitutions and altered codon usage frequencies is yet to be investigated by functionally expressing these (harmonized) genes with microorganisms. The latter will require a multidisciplinary approach and, more importantly, a huge effort towards standardization of DNA parts, experimental procedures and conditions, and data processing. Although some efforts are being done, and e.g. biorepositories are of great importance in this regard, the path ahead is still very long.

## Methods

### Genetic dataset

As a proof of principle, the genetic dataset was limited to include only oxidoreductases and transferases, genes belonging to the enzyme classes EC 1.x.x.x and EC 2.x.x.x. While genes were selected at random, attention was given to certain criteria to ensure sufficient variation in the genetic dataset. First, the UniProt Annotation score [58] had to be classified as maximum. Second, genes with varying amounts of protein domains, such as metal binding domains and transmembrane domains, were selected. Finally, the natural hosts of the selected genes were chosen from as many biological kingdoms (animals, plants, fungi, protista, monera) as possible. The gene dataset is given in Supplementary Table 2.

### Validation dataset

To validate the findings from statistical analysis on the genetic dataset, a new validation dataset was made. This dataset, just like the genetic dataset, also only comprises genes from the oxidoreductase and transferase enzyme families. For this dataset, genes were chosen that have academic or industrial relevance, while also checking for enough variability when it comes to GC content and secondary structures. To ensure this, two genes with high GC content and two with low GC content were chosen, while 4 genes with increasing secondary structures were selected as well. Finally, the natural hosts of the selected genes were chosen from as many biological kingdoms (animals, plants, fungi, protista, monera) as possible. The validation dataset is given in Supplementary Table 3.

### Codon harmonization tools

All genes listed in Supplementary Table 2 were harmonized using CodonWizard, EuGene, Galaxy and CHARMING (with modes %MinMax and Geometric Mean). When using CodonWizard, the genetic sequence was imported and harmonized with 0% tolerance to eliminate randomization effects in the results. Kazusa CUTs were used during both the harmonization and to compare the output with the original sequence, meaning that the use of the outdated Kazusa databases had no impact on the comparison. When using EuGene, both the target host’s and the natural host’s genome were imported into Gene Pool, and the gene of interest was manually added to the uploaded natural host’s genome. Importantly, the target host’s genome should be uploaded first as the drop-down menu allowing the user to choose which genome the genetic sequence should be harmonized to does not work properly. Large differences in harmonization results were observed when the order was reversed. Once the desired gene was added to the natural host’s genome, the gene was uploaded to the workspace, and harmonization (RSCU) was carried out without additional filters. A similar workflow was followed for Galaxy. First, both the heterologous and original host genomes were uploaded, and CUTs were calculated. Afterwards, the desired gene was harmonized toward the heterologous genome. When the online tool CHARMING was used, the gene sequence was imported, and the desired codon math for harmonization was selected among those available in the online tool. A window size of 17 was used. To limit the need for computational power, one harmonized output was requested. CUTs from Kazusa were used for data on the natural host’s codon usage. If the input sequence was longer than 350 codons, the CHARMING script was used (Python 3.9).

### Assessment of codon harmonization tools

To evaluate the efficacy of the various codon harmonization tools, %MinMax values [59] were calculated for a sliding window of 18 codons across the entire length of the gene. Afterwards, the differences between the %MinMax values of the harmonized sequence and those of the original sequence were calculated, obtaining a value called ΔMinMax. To evaluate the four tools and perform exploratory data analysis, the RMSE was calculated from ΔMinMax for each gene using the following formula:

$${\rm{RMSE}} = \sqrt {\sum _{i = 1}^n\frac{{{{({{{\rm{\hat y}}}_i} - {y_i})}^2}}}{n}}$$

with n = the number of sliding windows within a gene, y_i_ = the observed ΔMinMax for sliding window i and ŷ_i_ = the predicted ΔMinMax for sliding window i. The latter was set to zero, as each codon harmonization tool aims to nullify the codon usage bias between the original and the intended host.

Biological parameters GC-content, secondary structures, enzyme class and host were also included in the comparison. The GC content was calculated for each sliding window of 18 codons, while the %mRNA was determined by uploading the original gene to RNAfold [60] to calculate the percentage of each sliding window that was involved in secondary mRNA structures. The mean %GC and %mRNA were calculated by averaging the obtained values per gene.

To create the codon change heatmaps, a python script was written to calculate a percentage of occurrence for each codon both in all original genetic sequences (ORI%) and in all harmonized ones (HARM%). This percentage was calculated by dividing the number of occurrences of a certain codon by the total number of occurrences of all synonymous codons. A host-specific percentage of occurrence was calculated in a similar way from the CUTs of each host (both original (CUT_original_%) and heterologous (CUT_heterologous_%)) and was used to correct for the inherent differences in codon occurrences between different species. It makes the codon occurrences relative to a ‘theoretical’ value as a reference. Using these parameters, RMSE values were calculated for all codons for every tool-host combination as follows:

$${RMSE}_{codons}= \sqrt{{\sum }_{i=1}^{n}\frac{{(\left|ORI\%-{CUT}_{original}\%\right|- \left|HARM\%-{CUT}_{heterologous}\%\right|)}^{2}}{n}}$$

where n is the number of genes present in the dataset and thus 27.

The resulting RMSE_codons_ is a measure for the difference in CUT-corrected occurrence of a certain codon between the original sequence and the harmonized sequence. As before, lower RMSE values represent a better harmonization result for a certain codon. Finally, a codon heatmap was constructed by using these RMSE values. It visualizes which codons are harder to harmonize than others.

### Statistical analysis

Statistical analysis and figures were conducted and created using R Statistical Software (v4.2.2; R Core Team 2022) and its attached packages, such as the package ‘gee’ [38]. Due to the complexity of the data, the data were fit using GEE’s. In this way, the four different tools could be compared. In addition, the influence of various parameters (GC content, RNA folding, heterologous host and enzyme class) on codon harmonization accuracy could be assessed using GEEs combined with the Wald test. For each statistical analysis, significance was defined as a p value < 0.05.

### Electronic supplementary material

Below is the link to the electronic supplementary material.

Supplementary Material 1

## Data Availability

The full genetic dataset that was used in this study can be found in Supplementary Tables 2, alongside the needed information to find all required genomes.

## List of abbreviations

CAI: Codon Adaptation Index
CHARMING:Geo: CHARMING with mode Geometric mean
CHARMING:MM: CHARMING with mode %MinMax
CUT: Codon Usage Table
EC number: Enzyme Commission number
Eco: *Escherichia coli*
GEE: Generalized Estimating Equations
Geo: Geometric Mean
MM: %MinMax
MRMSE: Mean Root Mean Squared Error
MSE: Mean Squared Error
RMSE: Root Mean Squared Error
RSCU: Relative synonymous codon usage
Sce: *Saccharomyces cerevisiae*
