# Supplementary material for: An exploratory in silico comparison of open-source codon harmonization tools
Source: Microb Cell Fact. 2023 Nov 6;22:227. doi: 10.1186/s12934-023-02230-y (PMC10626681; doi:10.1186/s12934-023-02230-y)
Supplement: Supplementary file 1 — Supplementary Material 1 [file 12934_2023_2230_MOESM1_ESM.docx]

**An Exploratory *in silico* Comparison of Open-Source Codon Harmonization Tools**

Thomas Willems^1^, Wim Hectors^1^, Jeltien Rombaut^1^, Anne-Sofie De Rop^1^, Stijn Goegebeur^1^, Tom Delmulle^1^, Maarten L. De Mol^1^, Sofie L. De Maeseneire^1,^*, Wim K. Soetaert^1^

1 Centre for Industrial Biotechnology and Biocatalysis (InBio.be), Department of Biotechnology, Faculty of Bioscience Engineering, Ghent University, Coupure Links 653, 9000 Ghent, Belgium

* Correspondence: Sofie.DeMaeseneire@UGent.be


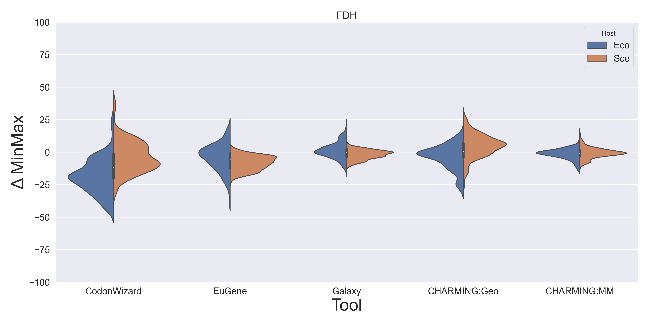

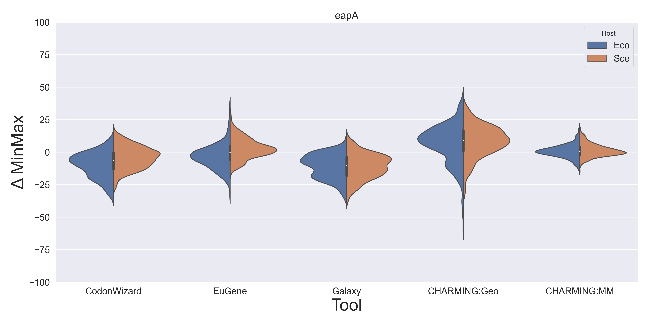

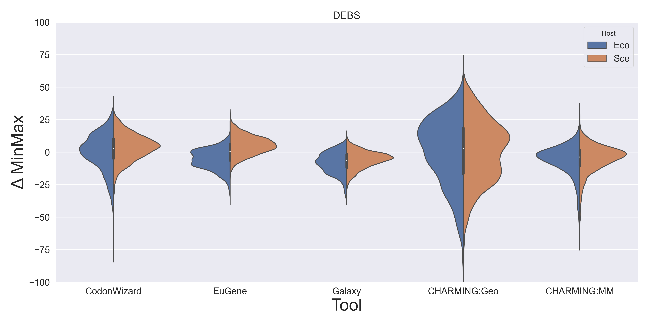

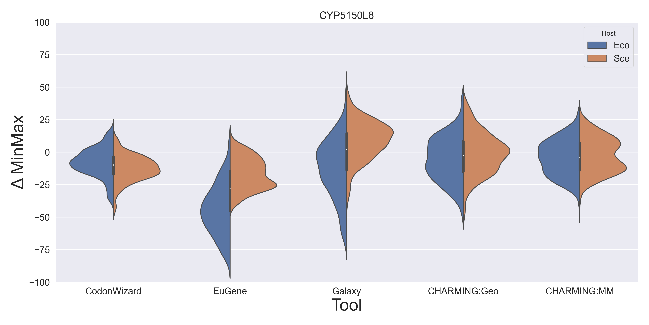

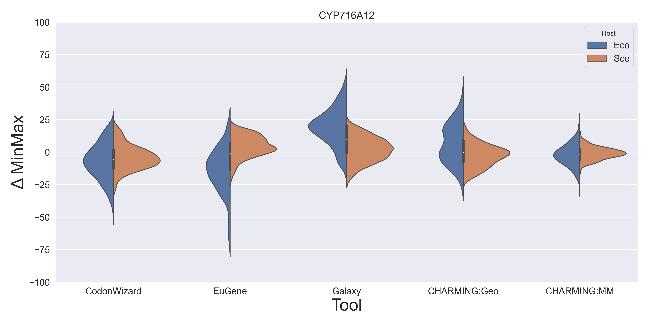

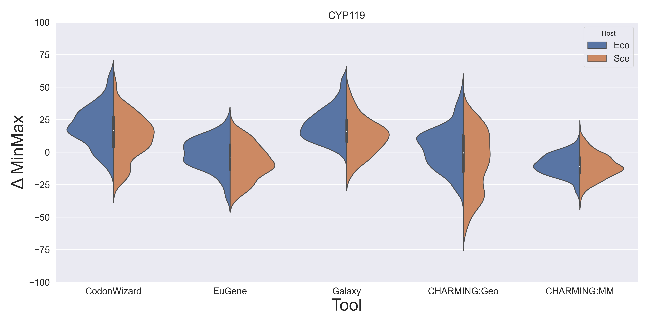

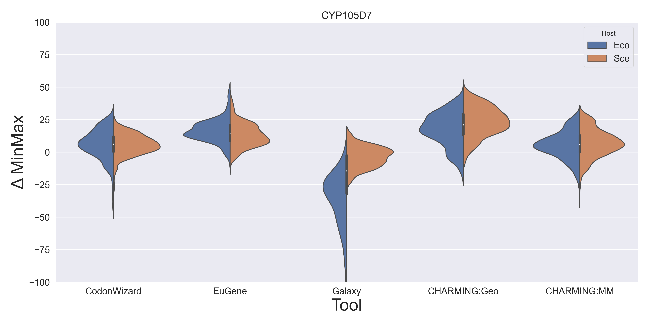

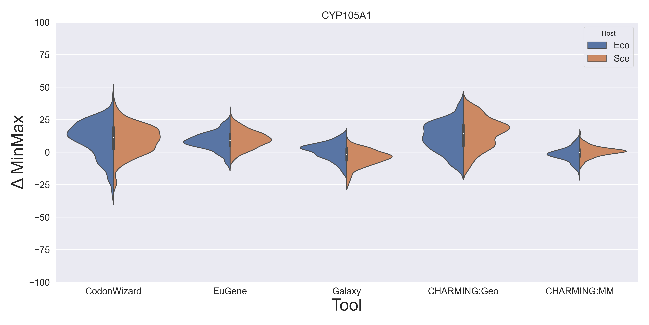

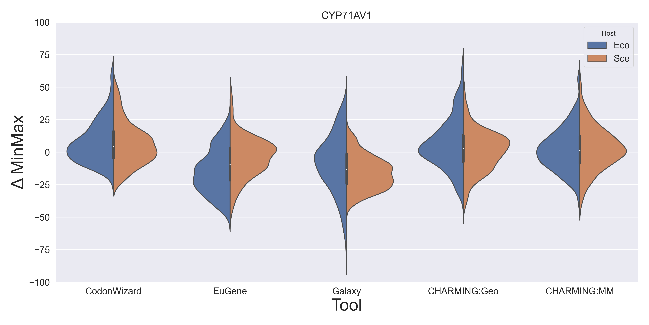

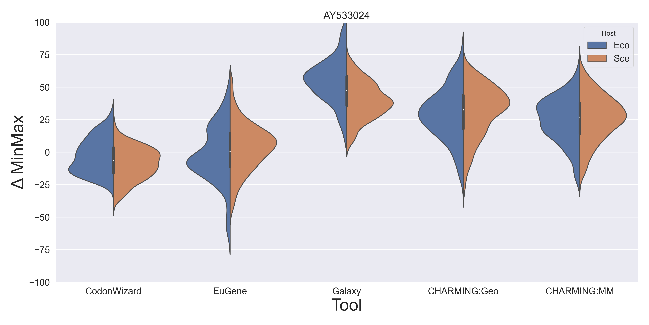

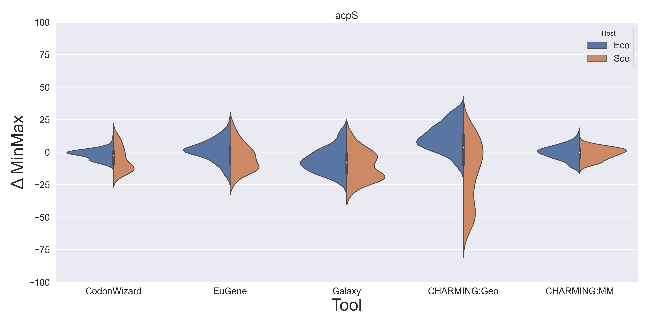

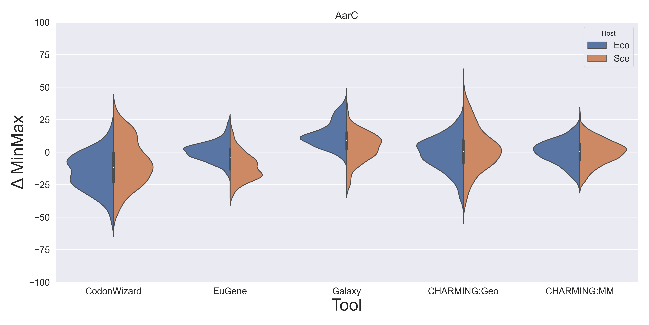


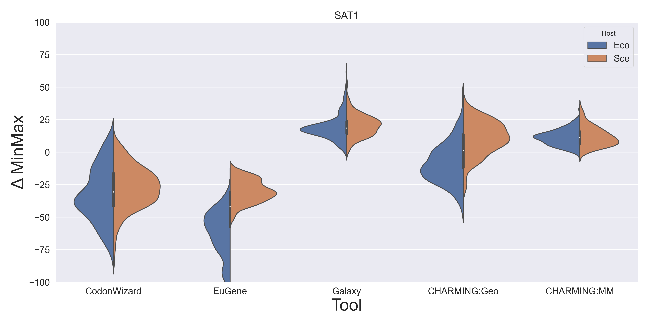

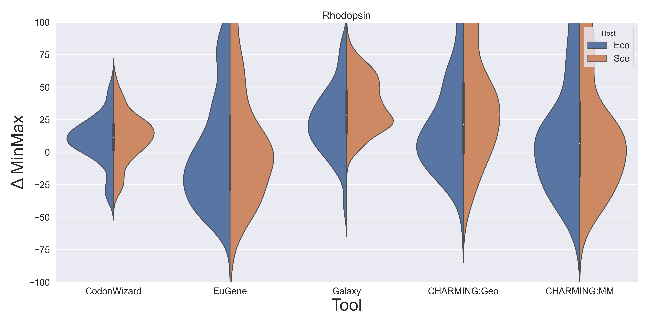

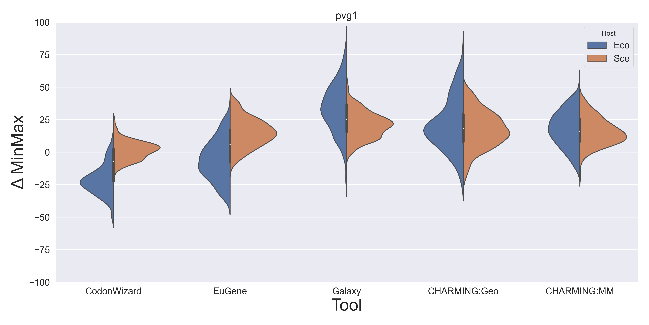

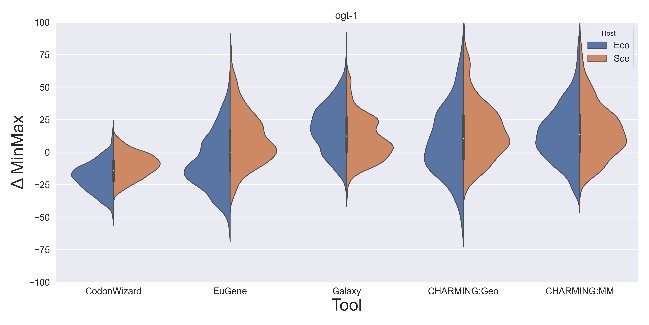

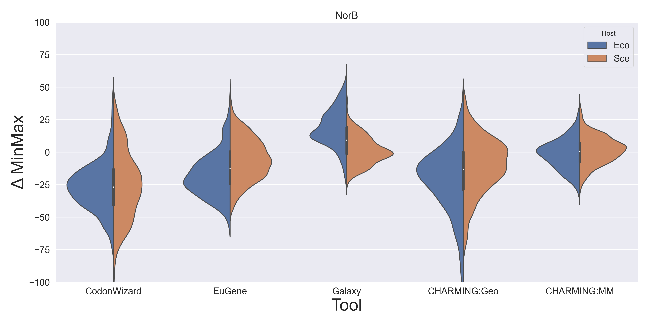

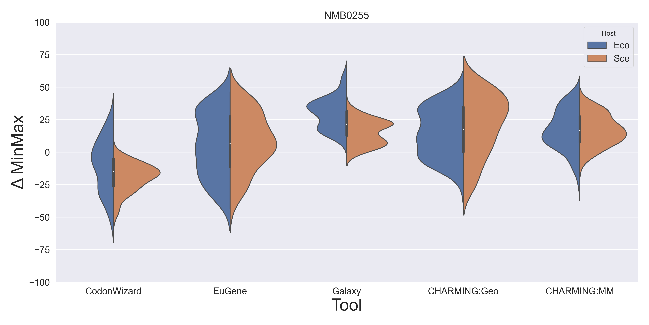

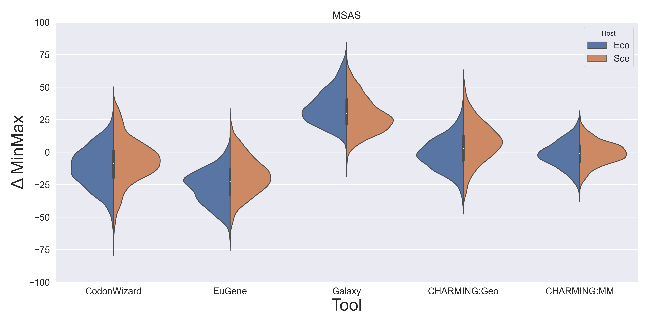

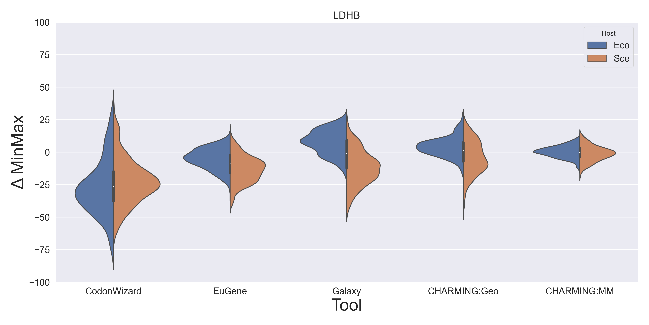

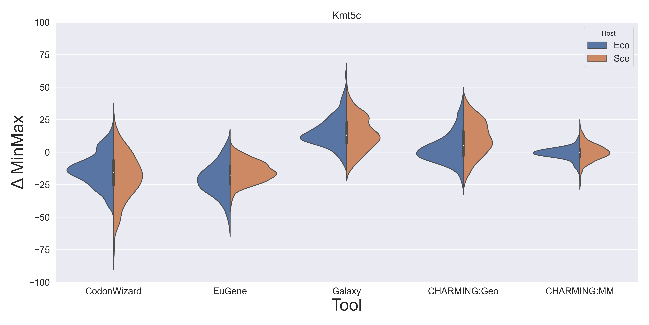

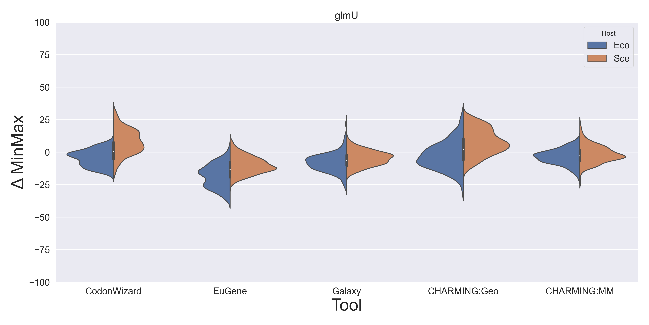

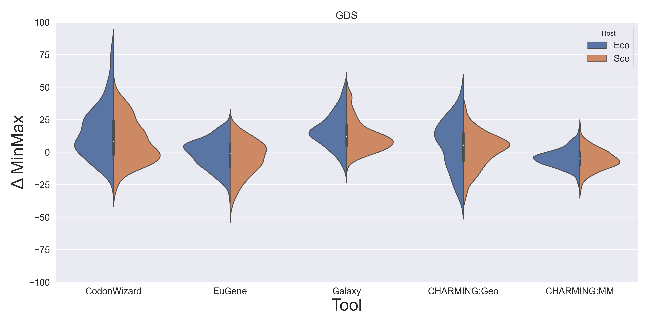

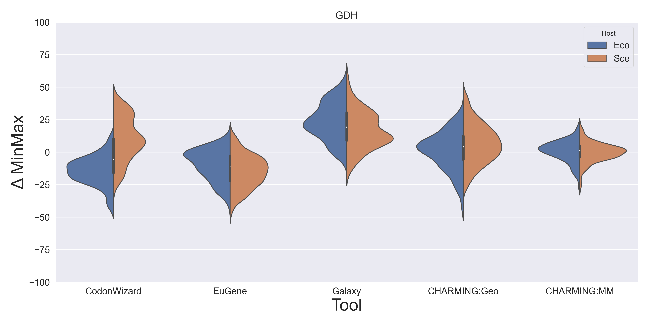


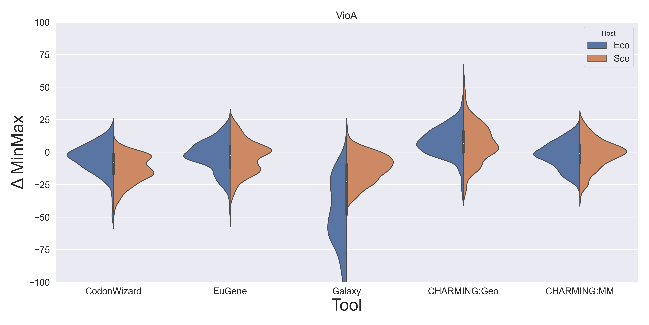

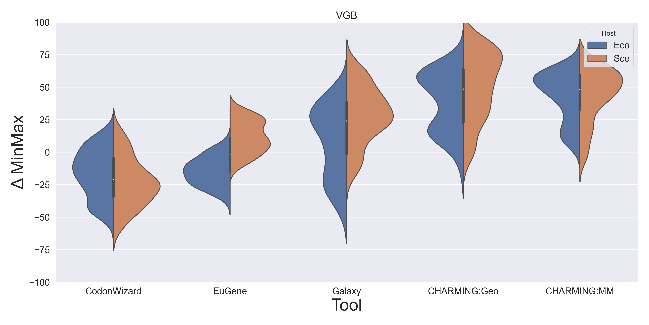

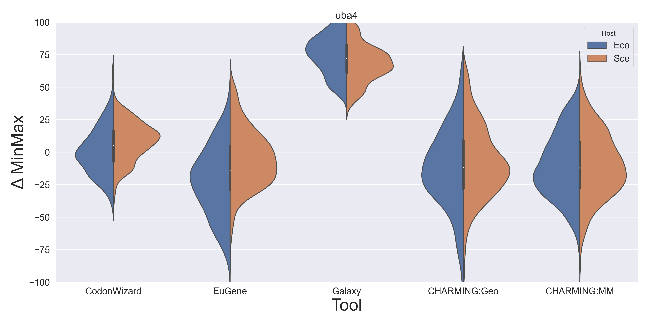


*Supplementary Figure 1: Violin plots of all the genes present in the gene dataset, in alphabetic order. In these plots, the difference in %MinMax between the codon usage of the harmonised gene and the original one is displayed as a distribution for each of the tools. (Escherichia coli = Eco and Saccharomyces cerevisiae = Sce).*

*Supplementary Table 1: A comparison of the various tools and their additional filters. The mRNA secondary structure filter optimizes mRNA stability, while the codon context filter accommodates for codon pair bias. The codon auto-correlation filter corrects for non-random distributions of consecutive codons, mainly at the beginning of the transcript and the repetition filter allows the user to predetermine the maximum amount of codons or bases that are allowed to be next to each other. The DNA motif filter allows the user to filter out any unwanted DNA motifs such as Shine-Dalgarno or restriction enzyme sites from the heterologous gene. Sometimes, a custom GC-content is desired, when for example the heterologous host is known to have an extremely low/high %GC. In order to optimize the translation efficiency of the synthetic gene, it could be wise to decrease the amount of hidden stop codons and/or take into regard that during translation, amino acid starvation could occur in the occurrence of too many similar consecutive codons. The final filters are a total number filter which allows the user to randomly distribute codons across the heterologous sequence according to the codon frequency table, or the use of a custom weight filter, meaning custom weights can be given to specific codons when the other filters are not able to properly interpret the intentions of the user.*

| **Gene Harmonization Tool** | **mRNA secondary structure** | **Codon context** | **Codon auto-correlation filter** | **Repetition filter** | **DNA motif**  **filter** | **GC/AT-content filter** | **Hidden stop codon filter** | **Amino acid starvation filter** | **Total number filter** | **Custom weight filter** |
| --- | --- | --- | --- | --- | --- | --- | --- | --- | --- | --- |
| **EuGene** | Yes | Yes | Yes | Yes | Yes | Yes | Yes | No | No | No |
| **CodonWizard** | No | No | No | Yes | No | Yes | No | Yes | Yes | Yes |
| **Galaxy** | No | No | No | No | No | No | No | No | No | No |
| **CHARMING** | No | No | No | No | No | No | No | No | No | No |

*Supplementary Table 2: Gene dataset used in this study. All genes are listed below, along with their Gene/GenBank/RefSeq ID, enzyme class, original host and Genome ID.*

| Gene | ID | Enzyme class | Organism | Genome ID |
| --- | --- | --- | --- | --- |
| AarC | GenBank: D13291.1 | Transferase | *Acetobacter aceti* | GCF_000379545.1 |
| acpS | Gene ID: 3920931 | Transferase | *Staphylococcus aureus* | GCF_000013425.1 |
| AY533024 | GenBank: AY533024.1 | Transferase | *Humulus lupulus* | GCA_023660075.1 |
| CYP71AV1 | GenBank: AB706288.1 | Oxidoreductase | *Artemisia annua* | GCA_014162995.1 |
| CYP105A1 | GenBank: CP029078.1, locus tag “DDJ31_35615” | Oxidoreductase | *Streptomyces griseoviridis* | GCF_003994395.1 |
| CYP105D7 | GenBank: AP019621.1, locus tag “SAVMC3_84600” | Oxidoreductase | *Streptomyces avermitilis* | GCF_000009765.2 |
| CYP119 | GenBank: U51337.1 | Oxidoreductase | *Sulfolobus acidocaldarius* | GCF_002215565.1 |
| CYP716A12 | GenBank: DQ335781.1 | Oxidoreductase | *Medicago truncatula* | GCF_003473485.1 |
| CYP5150L8 | Genbank: MF175172.1 | Oxidoreductase | *Ganoderma lucidum* | GCA_019426095.1 |
| DEBS3 | GenBank: AY661566.1 | Transferase | *Saccharopolyspora erythraea* | GCF_000062885.1 |
| eapA | GenBank: AJ010740.1 | Transferase | *Dictyostelium discoideum* | GCF_000004695.1 |
| FDH | Gene ID: 3877330 | Oxidoreductase | *Neurospora crassa* | GCF_000182925.2 |
| GDH | Gene ID: 938261 | Oxidoreductase | *Bacillus subtilis* | GCF_000009045.1 |
| GDS | Gene ID: 14550623 | Transferase | *Sulfolobus acidocaldarius* | GCF_002215565.1 |
| glmU | GenBank: L42023.1, locus tag “HI_0642” | Transferase | *Haemophilus influenzae* | GCF_000931575.1 |
| Kmt5c | RefSeq: NM_001358044.1 | Transferase | *Mus musculus* | GCF_000001635.27 |
| LDHB | Gene ID: 281275 | Oxidoreductase | *Bos taurus* | GCF_002263795.2 |
| MSAS | GenBank: X55776.1 | Transferase | *Penicillium griseofulvum* | GCF_001561935.1 |
| NMB0255 | GenBank: AE002098.2 | Transferase | *Neisseria meningitidis* | GCF_008330805.1 |
| NorB | Gene ID: 66586386 | Oxidoreductase | *Moraxella catarrhalis* | GCF_002080125.1 |
| ogt-1 | RefSeq: NM_001047395.5 | Transferase | *Caenorhabditis elegans* | GCF_000002985.6 |
| pvg1 | Gene ID: 2543508 | Transferase | *Schizosaccharomyces pombe* | GCF_000002945.1 |
| Rhodopsin | Gene ID: 13287905 | Oxidoreductase | *Leptosphaeria maculans* | GCF_000230375.1 |
| SAT1 | Gene ID: 4324655 | Transferase | *Oryza sativa subsp. Japonica* | GCF_001433935.1 |
| uba4 | RefSeq: XM_001259641.1 | Transferase | *Neosartorya fischeri* | GCF_000149645.3 |
| VGB | GenBank: AF292694.1 | Oxidoreductase | *Vitreoscilla sp.* | GCF_002951955.2 |
| VioA | GenBank: AF172851.1 | Oxidoreductase | *Chromobacterium violaceum* | GCF_016890085.1 |
|  |  |  |  |  |

*Supplementary Table 3: Validation dataset used in this study. All genes are listed below, along with their Gene/GenBank/RefSeq ID, original host and Genome ID.*

| Gene | ID | Organism | Genome ID |
| --- | --- | --- | --- |
| LDH | GenBank: M93720.1 | *Plasmodium falciparum* | GCA_000002765 |
| GSTF1 | GenBank: GQ377241.1 | *Populus trichocarpa* | GCF_000002775.5 |
| Opine dehydrogenase | GenBank: CP001734.1 | *Desulfohalobium retbaense* | GCF_000024325.1 |
| MnP-1 | GenBank: M60672.1 | *Phanerochaeta chrysosporium* | GCA_001910725.1 |
| sqr | GenBank: AE000657.1 | *Aquifex aeolicus* | GCF_000008625.1 |
| QR2 | GenBank: AF304462.1 | *Triphysaria versicolor* | PRJNA708087 |
| waaA | GenBank: Z31593.1 | *Chlamydia pneumoniae* | GCF_000007205.1 |
| easE | GenBank: AAHF01000001.1 | *Aspergillus fumigatus* | GCF_000002655.1 |
